# Negevirus Piura Suppresses Zika Virus Replication in Mosquito Cells

**DOI:** 10.3390/v16030350

**Published:** 2024-02-24

**Authors:** Valéria L. Carvalho, Dhani Prakoso, Erika R. Schwarz, Tracey D. Logan, Bruno Tardelli Diniz Nunes, Sarah E. Beachboard, Maureen T. Long

**Affiliations:** 1 Department of Arbovirology and Hemorrhagic Fevers, Evandro Chagas Institute, Ministry of Health, Rodovia BR-316, Km 7, s/n, Ananindeua 67030-000, PA, Brazil; 2 Professor Nidom Foundation, Surabaya, East Java 60236, Indonesia; dhaniprakoso@yahoo.com; 3 Montana Veterinary Diagnostic Laboratory, 1911 W Lincoln St., Bozeman, MT 59718, USA; 4 Department of Environmental and Global Health, College of Public Health and Health Professions, University of Florida, 1225 Center Dr. Suite 4101, Gainesville, FL 32611, USA; 5 Department of Comparative, Diagnostic, and Population Medicine, College of Veterinary Medicine, University of Florida, 1945 SW 16th Ave., Gainesville, FL 32608, USA; 6 Emerging Pathogens Institute, University of Florida, 2055 Mowry Road, Gainesville, FL 32610, USA

**Keywords:** Zika virus, Piura virus, coinfection

## Abstract

We investigated the interaction between the insect-specific virus, Piura virus (PIUV), and the arbovirus Zika virus (ZIKV) in *Aedes albopictus* cells. We performed coinfection experiments in C6/36 cells. Piura virus (Cor 33 strain, Colombia) and ZIKV (PRVABC58 strain, Puerto Rico) were co-inoculated into C6/36 cells using two multiplicity of infection (MOI) combinations: 0.1 for both viruses and 1.0 for ZIKV, 0.1 for PIUV. Wells were infected in triplicate with either PIUV and ZIKV coinfection, ZIKV-only, or PIUV-only. Mock infected cells served as control wells. The cell suspension was collected daily 7 days post-infection. Zika virus load was titrated by TCID_50_ on Vero 76 cells. The ZIKV-only infection and PIUV and ZIKV coinfection experiments were also quantified by RT-qPCR. We also investigated whether ZIKV interfered in the PIUV replication. PIUV suppressed the replication of ZIKV, resulting in a 10,000-fold reduction in ZIKV titers within 3 days post-infection. PIUV viral loads were not reduced in the presence of ZIKV. We conclude that, when concurrently infected, PIUV suppresses ZIKV in C6/36 cells while ZIKV does not interfere in PIUV replication.

## 1. Introduction

Arboviruses are viruses transmitted to vertebrates that may cause disease in humans and animals, leading to outbreaks and epidemics. Dengue (DENV), Zika (ZIKV), and chikungunya (CHIKV) viruses are primarily adapted to humans, and these three viruses have extended their geographic range due to the dissemination of *Ae. aegypti* and adaptation to the ubiquitous *Ae. albopictus* [1,2]. ZIKV, of the family *Flaviviridae*, genus *Orthoflavivirus*, caused several outbreaks in the Pacific Islands between 2007 and 2014 and, starting in 2015, causing an epidemic of febrile illness associated with microcephaly in infants and Guillain–Barré Syndrome in many countries. With transmission reported in 86 countries, ZIKV poses an ongoing threat to the developing fetus by causing neurological impairment with congenital infection (congenital ZIKV syndrome) [1,2,3,4]. Although cases of ZIKV congenital syndrome are rare at present, the virus is still circulating and there are no approved therapeutics or vaccines to treat or prevent the infection. Given the ever-expanding range of *Ae. Aegypti* and *Ae. Albopictus* and the increased frequency of outbreaks of anthrophilic arboviruses, it is important to maintain active surveillance and develop interventional strategies for the control of outbreaks [2].

Insect-specific viruses (ISVs) are a diverse group of viruses that naturally infect hematophagous insects, such as mosquitoes, ticks, phlebotomine sandflies, and others. Unlike arboviruses, ISVs do not appear to replicate in vertebrates and their cells but instead infect only invertebrates [5,6]. ISVs are mainly transmitted vertically and may share the same mosquito vector with arboviruses. Some ISVs, mainly those belonging to the viral families *Togaviridae* and *Flaviviridae*, are phylogenetically related to certain arboviruses, indicating that ISVs may be the ancestors of these arboviruses, which then gain the capacity to infect vertebrates, or vice versa [5,6].

While some ISVs are more phylogenetically related to arboviruses, others are closer to plant viruses, such as those belonging to the taxon *Negevirus* and the family *Tymoviridae* [7,8]. The taxon *Negeviruses* comprises several viruses, which are classified into two genera: *Nelorpivirus* and *Sandewavirus* [9]. Negeviruses replicate well in mosquito and sandfly cell lines, reaching high titer levels, demonstrating that these viruses are well adapted to these insect cells [10]. Phylogenetically, negeviruses are more related to plant-infecting viruses of the genera *Cilevirus*, *Higrevirus,* and *Blunervirus*. In addition to these ISVs demonstrating phylogenetic similarities to plant viruses, viruses have recently been isolated from plants that are more similar to *Negeviruses* or “nege-like”; future work with these and yet-to-be-discovered negeviruses will contribute to our understanding of the evolution of these viruses [10,11,12,13,14]. Furthermore, metagenomics conducted on the mosquito virome will likely identify new ISVs, as well as add to our knowledge of the worldwide distribution of these viruses [10,15].

One of the viruses belonging to the genus *Nelorpivirus* is Piura virus (PIUV), which was first isolated from *Culex* sp. mosquitoes in the city of Piura in Peru in 1996. In subsequent years, PIUV was detected in Mexico, Colombia, and the United States. The virus has been isolated from hematophagous insects of different genera, such as *Anopheles*, *Psorophora*, *Aedes*, *Uranotaeneia* and *Lutzomyia*. PIUV has three proteins, encoded in three open reading frames (ORF) called ORF 1, ORF 2, and ORF 3. Phylogenetically, PIUV is more related to Brejeira virus, which was isolated in Brazil [10].

Recent studies have investigated how ISVs and arboviruses interact within insects. This previous work demonstrated that certain ISVs, mainly orthoflaviviruses and alphaviruses, inhibit arbovirus replication in cells and reduce vector competence [16,17,18,19]. Bolling and colleagues (2012) conducted experiments using a laboratory colony of *Culex pipiens* mosquitoes naturally infected with Culex flavivirus (CxFV). The mosquitoes were infected with West Nile virus (WNV), and then the authors evaluated the mosquitoes’ vector competence, indicating that the suppression of WNV replication was caused by the persistent previous infection with CxFV.

This observation that ISVs modulate the replication of arboviruses highlights their potential as a future tool in the biological control of these viruses. ISVs have been used as the backbone for the development of vaccines against arboviruses. In particular, a candidate Chikungunya vaccine, using the Eilat virus as a backbone, was 100% protective in two different mouse models and blocked viremia in infected nonhuman primates [20]. Other chimeras developed with this backbone include the inclusion of structural genes from Eastern Equine Encephalitis virus (EEEV) and Venezuelan equine encephalitis virus (VEEV) [17]. These ISV chimeras also have potential for use as diagnostic reagents, allowing for work on highly pathogenic reagents at biosafety level 1 [21,22].

Despite studies showing the potential biotechnological use of orthoflaviviruses and alphaviruses as ISVs, little is known about negeviruses’ and arboviruses’ interaction. One recently published study showed that the negeviruses Negev (NEGV) and PIUV inhibited VEEV virus and CHIKV replication in mosquito cells [23]. To further understand the interaction of PIUV with arboviruses, we investigated the interaction between the PIUV and Zika virus (ZIKV) in *Ae. albopictus* cells. We hypothesized that, like the interaction observed between ISV and alphaviruses, PIUV would inhibit the replication of vertebrate viruses. To this end, we investigated the interactions between Piura and Zika viruses in vitro.

## 2. Materials and Methods

### 2.1. Cells and Viruses

African green monkey kidney cells (Vero 76) (ATCC, Manassas, VA, USA) and *Ae. albopictus* cells (clone C6/36) (ATCC, Manassas, VA, USA) were used for viral infections. Vero 76 cells were cultured in media consisting of advanced DMEM (Gibco, Waltham, MA, USA), supplemented with 5% Fetal Bovine Serum (FBS), 1% Hepes, 1% Penicillin/Streptomycin, 0.5% amphotericin and maintained at 37 °C in 5% CO_2_. The C6/36 cells were cultured in DMEM (1×) (Gibco, Waltham, MA, USA), supplemented with 10% FBS, 1% Glutamine, 1% Sodium Pyruvate, 1% Tryptose Phosphate Broth, 1% MEM Non-Essential amino acids, 1% Penicillin/Streptomycin, 0.5% amphotericin. C6/36 cells were maintained at 28 °C in 5% CO_2_.

ZIKV (strain PRVABC59, accession number KU501215.1) and PIUV (strain Cor 33, accession number KX518787.1) (both at passage 3) were grown in Vero 76 and C6/36, respectively. The cell suspension was harvested when an approximately 80% cytopathic effect (CPE) was observed. The cell suspension deriving from ZIKV and PIUV stock was concentrated using a Centricon Plus-70 Centrifugal Filter (Millipore, Burlington, MA, USA). Virus infection was titrated by TCID_50_ for ZIKV and PIUV on Vero 76 cells and C6/36 cells, and ZIKV and PIUV titers were 10^7^ TCID_50_/mL and 10^10^ TCID_50_/mL, respectively [24].

### 2.2. Coinfection of ZIKV and PIUV

Confluent C6/36 cells seeded in 24-well plates were inoculated with PIUV and ZIKV, PIUV only, or ZIKV only. We used two MOI combinations, consisting of 0.1 for both viruses and then a combination of 1.0 for ZIKV and 0.1 for PIUV. Wells were inoculated in triplicate in the C6/36 cells. Negative controls consisted of mock-challenged cells. The cell suspension was collected daily 7 days post-infection and frozen at −80 °C. ZIKV viral load was determined for each well using a TCID_50_ performed in Vero 76 cells to determine if PIUV interfered in ZIKV replication.

### 2.3. Tissue Culture Infectious Dose 50% (TCID_50_) 

Vero 76 cells were seeded in 48-well plates. When the cells were confluent, the media was removed and 100 μL of 10× serial dilutions were inoculated into four wells for each dilution. The plates were incubated at 37 °C for one hour; plates were rocked every 15 min to distribute the virus across the cell monolayer. After incubation, 0.5 mL of advanced DMEM maintenance media with 2% FBS was added to each well. The plates were incubated at 37 °C and monitored for CPE for seven days.

On day 7 post-inoculation, the plates were fixed using cold 25% methanol for 10 min. The methanol was removed, and the cells were stained with 0.5% crystal violet and incubated at room temperature for 30 min. The plates were rinsed in water and then allowed to dry. The CPE was graded in each well and the TCID_50_ was calculated using the Reed–Muench method (1938) [24].

### 2.4. Intracellular Replication

To assess the effect of PIUV on ZIKV intracellular replication, cells were co-infected with PIUV and ZIKV, both at an MOI of 0.1, in confluent C6/36 cells. These viruses were inoculated in triplicate; mock-challenged wells and positive controls were also included. Cells were collected every 24 h post-inoculation for 72 h. At each timepoint, the supernatant was removed, the cells were washed with PBS, and then guanidinium–isothiocyanate-chloroform (TRIzol Reagent, Invitrogen, Carlsbad, CA, USA) was added directly to the cells. The cells were scraped and RNA was extracted as previously described [25]. The RNA was quantified in each sample by spectrophotometry (Nanodrop, Thermo, MA, USA). Real-time RT-qPCR was used to compare viral loads.

Indirect immunofluorescence was used to detect viruses in the cells. For this, we seeded C6/36 cells into culture slides (Millicell EZ Slide, Millipore, Burlington, MA, USA) and inoculated the cells with the viruses as previously described. Cells were fixed every 24 h post-inoculation for 72 h. After fixation, anti-orthoflavivirus antibody 4G2 was applied to each well and incubated for 30 min at 28 °C. The 4G2 antibody was purified from D1-4G2-4-15 hybridoma cells (ATCC #HB-112, cultured in RPMI medium with 10% FBS). After washing, goat anti-mouse IgG Fc-FITC (Southern Biotech, Birmingham, AL, USA) was added to each well. Finally, the cells were stained with DAPI (BD Pharmingen, San Diego, CA, USA) and read using a fluorescence microscope (all-in-one, BZ-X810) (Keyence, Osaka, Japan).

### 2.5. ZIKV and PIUV RNA Quantification

ZIKV RNA from the ZIKV-only and PIUV/ZIKV coinfection wells were tested by RT-qPCR one-step protocol for ZIKV using the forward primer (5′ CCGCTGCCCAACACAAG 3′), reverse primer (5′ CCACTAACGTTCTTTTGCAGACAT 3′) and probe (5′ FAM- AGCCTACCTTGACAAGCAGTCAGACACTCAA—BHQ-1 3′) previously described [26].

To investigate whether ZIKV interfered with PIUV replication, we developed an RT-qPCR for PIUV. First, we sequenced the PIUV full-length genome (Cor 33_TVP 20159 strain/Genbank accession number: KX518787) and used Primer3 software to design several PIUV primers and probes. Oligonucleotides were synthesized by LGC Biosearch Technologies (Petaluma, CA, USA). Primers were first optimized using SYBR green chemistry (Fast SYBR Green Master Mix; Applied Biosystems, Foster City, CA, USA). Once the primer specificity and concentrations of each were optimized against the PIUV Cor 33 strain, a probe-based system was developed (TaqMan, Applied Biosystems, Foster City, CA, USA).

Based on this initial optimization, the following primers and probes were utilized: the forward primer CO_Cor33-F1, ATCGGTGCGAATACCATAGC and reverse primer CO_Cor33-R1, GTTTACTCCTGACCCCGTGA. The probe sequence of CO_Cor33_Probe was FAM-ATCTTTCGTGGATACCGTGC-BHQ-1. The product length of the RT-PCR is 178 bp and the target is the ORF 1 (hypothetical protein 1). The total reaction volume was 20 μL, consisting of 1.6 μL forward primer (400 nM), 1.6 μL reverse primer (400 nM), 5 μL TaqMan Fast Virus 1-Step Master Mix, 1 μL probe (250 nM), 8.8 μL nuclease-free water, and 2 μL RNA template.

We developed a one-step RT-PCR protocol using the following cycling conditions: 5 min at 50 °C (reverse transcription) and 20 s at 95 °C (RT inactivation/initial denaturation), followed by 40 cycles of denaturation/anneal/extension (3 s at 95 °C and 30 s at 60 °C). To determine the reaction efficiency and linearity, a standard curve was prepared using 10-fold dilution (10-1 to 10-5) of the PIUV RNA (10^10^ TCID50/mL, 1.17 × 10^15^ copies of RNA) in triplicate. Our newly developed PIUV RT-qPCR assay showed an efficiency of 96%, a correlation coefficient (R2) of 0.996, and a slope value of −3.408.

### 2.6. Statistics

The TCID_50_ and RT-qPCR results were analyzed using a two-way ANOVA, using posteriori Bonferroni correction for multiple comparisons, using commercially available statistics software (GraphPad Prism 9.3.1).

## 3. Results

### 3.1. PIUV Causes Intense CPE in C6/36 Cells

In our initial experiments, we performed a pilot study with PIUV at different MOIs (0.1, 1, 3, 5) in C6/36 cells, and we observed that PIUV caused approximately 90% of CPE at day 1 post-infection at all MOIs; thus, a co-infection experimental design was pursued. Suprainfection was not possible due to the highly cytopathic nature of PIUV in C6/36 cells.

### 3.2. PIUV Inhibits ZIKV Replication in C6/36, but ZIKV Does Not Interfere with PIUV Replication

**Figure 1:**
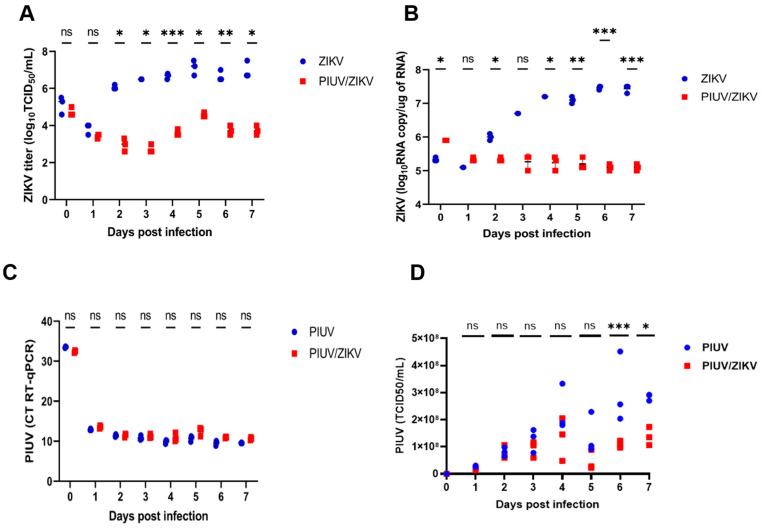
PIUV reduces ZIKV titer in C6/36 cells (ZIKV and PIUV both at MOI 0.1). (**A**) ZIKV TCID_50_ titers in the “ZIKV-only” and “ZIKV and PIUV coinfection” wells. (**B**) ZIKV RNA copy number “ZIKV-only” and “ZIKV and PIUV coinfection” wells. (**C**) Threshold cycle (CT) of PIUV (RT-qPCR), comparing “PIUV-only” and “ZIKV and PIUV coinfection” wells. (**D**) PIUV TCID_50_ titers in the “PIUV only” and “ZIKV and PIUV coinfection” wells. ns: not significant (*p* ≥ 0.05); *: significant (*p* 0.01 to 0.05), **: very significant (*p* 0.001 to 0.01), ***: extremely significant (*p* 0.0001 to 0.001).

To determine if PIUV interferes with ZIKV replication and vice versa, we performed coinfection experiments in C6/36 cells using MOI 0.1 for both viruses. Our results showed that, when PIUV is present, ZIKV replication is suppressed by up to a 10,000-fold reduction in titers by three days post-infection (*p* < 0.0001) (Figure 1A). The RT-qPCR also showed a reduction in ZIKV viral load in the coinfection samples (*p* < 0.0001) (Figure 1B). PIUV viral loads (cycle threshold, Ct) were not reduced in the co-infection wells compared to the PIUV-only wells (Figure 1C). The PIUV titer (TCID_50_) also showed no significant reduction in most time points (Figure 1D).

### 3.3. PIUV Inhibition of ZIKV Replication Is Dose-Dependent

**Figure 2:**
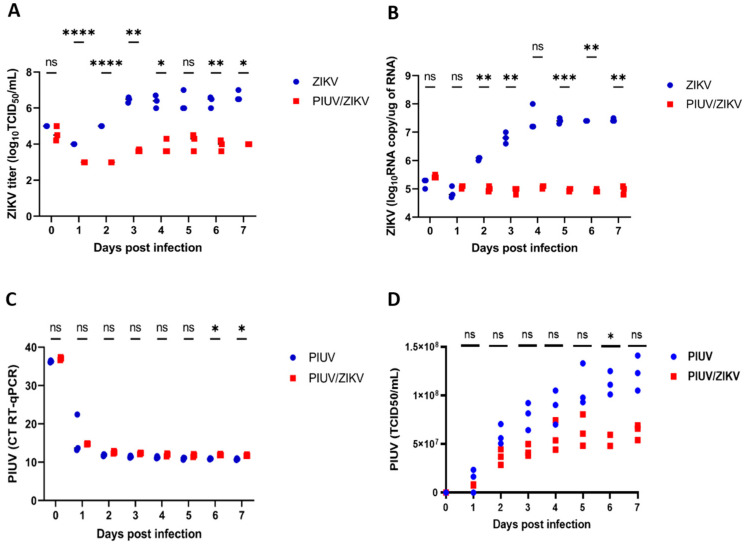
PIUV reduces ZIKV titer in C6/36 cells (ZIKV at MOI 1 and PIUV at MOI 0.1). (**A**) ZIKV TCID_50_ titers in “ZIKV-only” and “ZIKV and PIUV coinfection” wells. (**B**) ZIKV RNA copy number in “ZIKV-only” and “ZIKV and PIUV coinfection” wells. (**C**) Threshold cycle (CT) of PIUV (RT-qPCR), comparing “PIUV-only” and “ZIKV and PIUV coinfection” wells. (**D**) PIUV TCID_50_ titers in the “PIUV only” and “ZIKV and PIUV coinfection” wells. ns: not significant (*p* ≥ 0.05); *: significant (*p* 0.01 to 0.05), **: very significant (*p* 0.001 to 0.01), ***: extremely significant (*p* 0.0001 to 0.001), ****: extremely significant (*p* < 0.0001).

To determine if ZIKV cultivated at a greater MOI is inhibited by PIUV, we performed co-infection experiments with ZIKV at an MOI of 1 and PIUV at an MOI of 0.1. Our results showed that PIUV inhibition of ZIKV was sustained, which resulted in a 1000-fold reduction in ZIKV titers by three days post-infection (*p* < 0.0001) (Figure 2A). While the inhibition was less at a 10× higher dose, the difference was still significant. Viral transcripts of ZIKV were also significantly reduced in the coinfection samples (*p* < 0.0001) (Figure 2B). On the other hand, PIUV viral loads, as detected by RT-qPCR, were not reduced in the co-infection wells compared to the PIUV-only wells (most of the time points were non-significant) (Figure 2C), in accordance with the PIUV TCID_50_ titers that were not significantly reduced in the presence of the ZIKV in almost all time points (Figure 2D). These findings indicate that the interference of PIUV in ZIKV replication is dose dependent.

### 3.4. PIUV CPE Was Predominant in PIUV/ZIKV Coinfection

**Figure 3:**
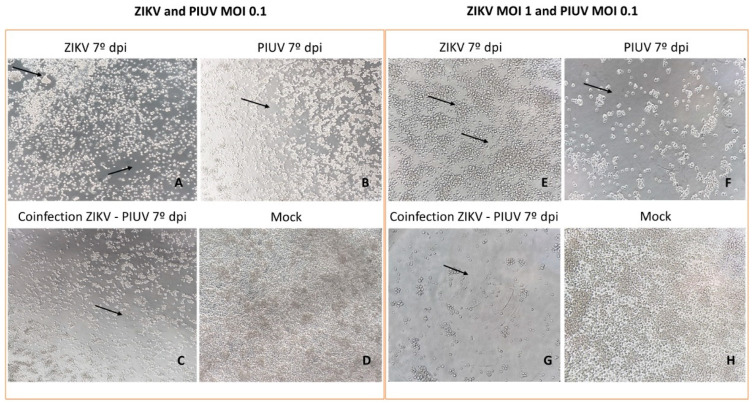
Infected C6/36 cells showing cytopathic effect (CPE) (black arrow) and mock-challenged cells. (**A**–**D**) ZIKV and PIUV MOI 0.1. (**A**) Cells infected with ZIKV (7° dpi.), presenting more prominent clumps of cells and cell destruction (100×). (**B**) Cells infected with PIUV (7° dpi.), showing considerable dead cells and spaces between cells (100×). (**C**) Cells coinfected with ZIKV and PIUV (7° dpi.), displaying the evolution of the cell death and destruction of the monolayer (100×). (**D**) Mock-challenged cells (100×). (**E**–**H**) ZIKV MOI 1 and PIUV MOI 0.1. (**E**) Cells infected with ZIKV (7° dpi.) (showing the evolution of the CPE) (200×). (**F**) Cells infected with PIUV (7° dpi.) (showing the evolution of the CPE) (200×). (**G**) Cells coinfected with ZIKV and PIUV (7° dpi.) (showing the evolution of the CPE) (200×). (**H**) Mock-challenged cells (200×).

Both viruses caused CPE in C6/36 cells. ZIKV caused the formation of clumps of cells with consecutive cell destruction. PIUV induced rapid cell death, characterized by a decrease in cell size and destruction of the cell monolayer. The predominant CPE visualized in co-infected C6/36 cells was that of PIUV, which consisted of rapid cell shrinkage and cellular death without clumping by the first day of infection (Figure 3).

### 3.5. PIUV Does Not Inhibit ZIKV Cellular Entry and Likely Inhibits Intracellular Replication in C6/36 Cells

**Figure 4:**
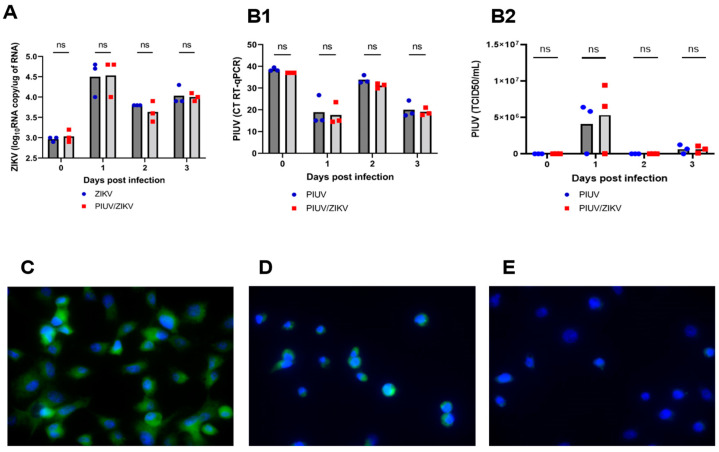
PIUV does not inhibit ZIKV cellular entry but inhibits intracellular replication in C6/36 cells (ZIKV and PIUV at MOI 0.1). Investigation of the intracellular replication: (**A**) ZIKV RNA copy number in the condition “ZIKV-only” and “ZIKV and PIUV coinfection”. (**B1**) Threshold cycle (CT) of PIUV (RT-qPCR), comparing the conditions of “PIUV-only” and “ZIKV and PIUV coinfection”. (**B2**) PIUV TCID_50_ titers in the “PIUV only” and “ZIKV and PIUV coinfection” wells. (**C**) Positive Indirect Immunofluorescence using 4G2 anti-orthoflavivirus antibodies: ZIKV (100×) (3° dpi.). (**D**) Positive Indirect Immunofluorescence using 4G2 anti-orthoflavivirus antibodies: ZIKV and PIUV coinfection (100×) (3° dpi.) (less fluorescence intensity in comparison with C). (**E**) Negative Indirect Immunofluorescence using 4G2 anti-orthoflavivirus antibodies: mock challenge (100×). ns: not significant.

We performed experiments to determine the stage at which PIUV interfered with ZIKV infection. To investigate whether PIUV interference occurred at the level of the intracellular replication of ZIKV, we examined cellular viral load every 24 h over 3 days by removing the supernatant, followed by rinsing the cells and adding TriZol directly to the cells (see Section 2.4). There was no difference in the ZIKV load (Ct) and TCID_50_ titers between cells infected only with ZIKV and those coinfected with ZIKV/PIUV, and in the PIUV load between cells infected with “PIUV-only” and those with ZIKV/PIUV coinfection (Figure 4A,B1,B2). The results of immunofluorescence using the anti-orthoflavivirus antibody (4G2) showed the presence of the ZIKV antigen in the cytoplasm of both cells infected only with ZIKV and ZIKV/PIUV co-infected cells; however, fluorescence intensity was greater in the cells infected only by ZIKV (Figure 4C–E).

## 4. Discussion

Our study showed that PIUV inhibits ZIKV replication when both viruses are simultaneously inoculated in C6/36 cells. These findings are consistent as ISVs likely have a long-term coexistence with mosquitoes and are well adapted to them, which may explain their replicative advantage compared to many arboviruses, which, hypothetically, may have evolved from ISVs. These results are promising since they provide initial data demonstrating that PIUV has potential as a biological control agent against ZIKV. This is one of the first studies showing that ISVs of different taxonomical groups, such as negeviruses, can inhibit arboviruses belonging to the family *Flaviviridae*. The fact that arboviruses and negeviruses did not present a genetic relationship and are structurally different favors the future use of ISVs of the taxon *Negevirus* as platforms in paratransgenic applications, decreasing the concerns about the evolution of ISV and its becoming a pathogenic virus, which is a limitation of the ISVs belonging to the *Flaviviridae* and *Togaviridae* families.

Our study is the first or one of the first to report the interference of a negevirus in the replication cycle of an arbovirus belonging to the family *Flaviviridae.* In our experiments, PIUV caused intense CPE in C6/36 cells 24 h post-infection, which precluded the ability to perform superinfection experiments. This intense pathogenicity has been shown by others, wherein Negeviruses reached very high titers in mosquito cells, such as C6/36 cells, with rapid cell death and loss of the monolayer [10,12]. In previous work, other ISVs, belonging mainly to the *Flaviviridae* and *Togaviridae* families, inhibited the replication of arboviruses from the same viral families. Limited information exists regarding the interaction between ISVs of the *Negevirus* genus and those from other arboviral families. A recent study showed, for the first time, that negeviruses such as Negev virus (NEGV) and PIUV strain EVG 7-47 (PIUV-Culex) (isolated from a pool of *Culex nigripalpus* mosquitoes from Florida, USA in 2013) induced superinfection exclusion of the arboviruses VEEV and CHIKV (*Togaviridae*, *Alphavirus*) in mosquito cells [23].

We performed coinfection experiments (concomitant infection) with PIUV and ZIKV in *Ae. albopictus* cells, each at an MOI of 0.1. PIUV suppressed ZIKV replication in these mosquito cells, as ZIKV titers decreased 10,000-fold in the presence of PIUV. Interestingly, we did not observe interference in the PIUV replication by ZIKV. Bolling and colleagues (2012), investigating the relationship of the CxFV and WNV, also observed that ISVs decreased WNV titers, while the WNV did not interfere with CxFV titers [16].

We observed that the inhibition in the ZIKV replication is dose-dependent even with a 10-fold higher MOI, as there was a significant reduction in the ZIKV titers, although less inhibition was present at the lower MOI of 0.1 for both viruses. Visually, we observed the dominance of PIUV CPE in the co-infection experiments; however, further studies of virus/cell infection are necessary to confirm this observation. The study performed by *Patterson* et al. [23] demonstrated that PIUV reduced CHIKV titers between 2.4 log_10_ and 5.3 log_10_ PFU/mL, also leading to significant reductions in the VEEV titers. Our results and those reported by Patterson et al. [23] show the heterologous interference of negeviruses, especially PIUV, in the replication cycle of arboviruses from the families *Flaviviridae* and *Togaviridae*. In other studies of homologous interference, Nhumirim virus (NHUV) of the *Flaviviridae* family inhibited DENV serotype 2 (DENV-2), ZIKV, and WNV by 4, 5, and 6 logs, respectively. No interference in NHUV replication occurred with co-infection [18,27,28]. Another study demonstrated that the ISV Palm Creek (*Orthoflavivirus*) interfered in WNV and Murray Valley encephalitis virus in vitro [29] and in WNV transmission in *Culex annulirostris* mosquitoes [30].

Once it was verified that PIUV inhibits the cellular replication of ZIKV, we investigated whether this interference occurred at the level of entry or beyond in the replication cycle. Our results showed that ZIKV still enters mosquito cells, suggesting that the interference is an intracellular event. These results point to the possible interference of the PIUV at some stage of replication other than genome synthesis since, intracellularly, the virus load between the ZIKV-only and the co-infection cells was similar. Since the viral load measured in the supernatant combined with cells was significantly less with co-infection, inhibition likely occurs during downstream events, such as ZIKV assembly, release, protein synthesis, or other stages.

Future studies must clarify which mechanisms are involved in the interference process and confirm these findings in vivo. In particular, given the defective RNA interference (RNAi) pathway in C6/36 cells, it is necessary to perform experimental infections in mosquitoes in vivo to analyze differences in the vector competence, even though many studies have shown concordance in the results obtained from in vitro and in vivo experiments. Different responses and pathways are involved in the immune system of mosquitoes, especially the RNAi, which is believed to be their primary antiviral mechanism. ISVs developed routes to escape the mosquito’s immune system, suppressing or blocking these pathways, mainly through RNAi suppressor molecules (VSRs) [31].

Further, although most of the studies show that several insect-specific viruses inhibit the replication of arboviruses in mosquitoes and their cells, each case must be evaluated carefully, considering the different species of mosquitoes, insects from different locations, and the virus strain, which may lead to different, and sometimes contradictory, results [30,32,33,34]. The study of Kent and colleagues (2010) [30] demonstrated that ISVs can be suppressed by co-infection with other viruses. The researchers observed that the ISV CxFV Izabal did not suppress WNV replication in C6/36 cells and *Culex quinquefasciatus* mosquitoes. Most importantly, WNV transmission was enhanced in CxFV-infected *Culex quinquefasciatus* mosquitoes from Honduras [30]. The mechanisms used by ISVs to inhibit arbovirus replication are still unclear, but one of the possibilities is that this occurs through superinfection exclusion, in which cells previously infected with one virus became unsusceptible to the second virus [35].

The studies of the interaction between the mosquito microbiota and arboviruses provide essential information to better understand their relationship and how we can use this in favor of public health. A good example is the bacteria *Wolbachia*, which has been used to control the transmission of arboviruses such as DENV and CHIKV through *Aedes aegypti* mosquitoes in endemic and epidemic areas [35,36,37]. More recently, ISVs have gained attention as potential biotechnological tools for the biological control of arboviruses, as platforms for vaccines, and as having potential use in the development of safe laboratory diagnostics. However, it is essential to understand the ISV-*Wolbachia* interaction, since both agents infect mosquitoes, and the presence of *Wolbachia* may lead to higher ISV titers or the opposite [35]. With this knowledge, other possibilities can be explored, such as combining *Wolbachia*-ISV to increase arbovirus inhibition [35]. In addition, understanding the virome of nonpathogenic viruses in mosquitoes can help in studies on mosquito ecology [38].

Our study demonstrates that ZIKV, an important virus in terms of public health, is inhibited by the ISV of the taxon *Negevirus*, PIUV, in mosquito cells in vitro. These findings may provide opportunities for the development of new tools to block or decrease virus transmission. Although the number of disease cases caused by ZIKV has decreased in recent years, the virus is still circulating at low levels, causing disease, and it is also spreading to new areas around the world [39]. In a recent study of the cumulative global ZIKV burden since 2015, ZIKV infections demonstrated an increasing trend from 2011 to 2015, followed by a decreasing trend [40]. Importantly, when events occur, women between the ages of 15 and 49 are most affected. This indicates that the potential for ZIKV’s congenital transmission still exists. Nonetheless, these years of low incidence provide an opportune moment to look for new tools to prevent, limit, or combat new outbreaks, and to prepare for the emergence of the virus in new areas with vulnerable populations.

## Data Availability

The original contributions presented in the study are included in the article, further inquiries can be directed to the corresponding author/s.
